# Integrated analyses of miRNAome and transcriptome reveal zinc deficiency responses in rice seedlings

**DOI:** 10.1186/s12870-019-2203-2

**Published:** 2019-12-26

**Authors:** Houqing Zeng, Xin Zhang, Ming Ding, Yiyong Zhu

**Affiliations:** 1 College of Life and Environmental Sciences, Hangzhou Normal University, Hangzhou, 311121 China; 2 College of Agriculture and Biotechnology, Hunan University of Humanities, Science and Technology, Loudi, 417000 China; 3 College of Resources and Environmental Sciences, Nanjing Agricultural University, Nanjing, 210095 China

**Keywords:** Rice (*Oryza sativa*), Zinc deficiency, microRNA, Transcriptome, Copper, Oxidative stress

## Abstract

**Background:**

Zinc (Zn) deficiency is one of the most widespread soil constraints affecting rice productivity, but the molecular mechanisms underlying the regulation of Zn deficiency response is still limited. Here, we aim to understand the molecular mechanisms of Zn deficiency response by integrating the analyses of the global miRNA and mRNA expression profiles under Zn deficiency and resupply in rice seedlings by integrating Illumina’s high-throughput small RNA sequencing and transcriptome sequencing.

**Results:**

The transcriptome sequencing identified 360 genes that were differentially expressed in the shoots and roots of Zn-deficient rice seedlings, and 97 of them were recovered after Zn resupply. A total of 68 miRNAs were identified to be differentially expressed under Zn deficiency and/or Zn resupply. The integrated analyses of miRNAome and transcriptome data showed that 12 differentially expressed genes are the potential target genes of 10 Zn-responsive miRNAs such as miR171g-5p, miR397b-5p, miR398a-5p and miR528-5p. Some miRNA genes and differentially expressed genes were selected for validation by quantitative RT-PCR, and their expressions were similar to that of the sequencing results.

**Conclusion:**

These results provide insights into miRNA-mediated regulatory pathways in Zn deficiency response, and provide candidate genes for genetic improvement of Zn deficiency tolerance in rice.

## Background

Zinc is one of the essential micronutrients which acts as a catalytic, regulatory or structural co-factor for a lot of enzymes and regulatory proteins in plants and animals [1]. The well-known Zn-containing proteins in plants include the enzymes like carbonic anhydrase, alcohol dehydrogenase and copper (Cu)/Zn superoxide dismutase (CSD), and lots of Zn-finger domain-containing proteins, which function in transcriptional regulation [1, 2]. Zn deficiency (−Zn) is a serious agricultural problem in arable soils worldwide due to the low availability of Zn that is caused by factors such as high pH, prolonged flooding, low redox potential, and high contents of bicarbonate, organic matter and phosphorus [3]. Zn deficiency in soils and plants can also result in human malnutrition through the intake of food that contains low concentrations of Zn and other micronutrients [4]. It is estimated that one-third of the human population, especially children and women suffer from Zn-deficiency-related health problems [5]. Thus, studies to better understand the mechanisms of Zn deficiency response and tolerance in plants can help to develop crops with increased Zn use efficiency, Zn concentration, and Zn deficiency tolerance [4, 6–8].

Rice (*Oryza sativa*) is the second most cultivated crop and the main staple food for three billion people in the world. However, Zn deficiency is becoming one of the most widespread soil constraints affecting rice productivity [9, 10]. Much effects have been made to elucidate the physiological and biochemical mechanisms associated with Zn deficiency response and tolerance in plants [11–13]. These mechanisms include increasing Zn availability for root uptake by adjusting the root system architecture, releasing of phytosiderophores and organic acids, and formation of arbuscular mycorrhiza symbiosis [6, 14, 15]; expanding the generation of crown roots [16]; involvement of transporters such as ZRT, IRT-related proteins (ZIPs), metal tolerance proteins and heavy metal tolerance family proteins in Zn uptake and translocation [17–21]; adjusting the expression of Zn-requiring enzymes [2, 13]. Recently, transcriptomic profilings were analyzed to identify Zn-responsive genes in plants [22–24]. Four genes up-regulated in Zn-efficient rice varieties under Zn deficiency were reported to be candidate genes conferring Zn efficiency [22]. A large number of Zn deficiency responsive genes were found to be associated with calcium, sugar and hormonal signals in soybean [23]. Although some progress has been made, the molecular regulatory mechanisms underlying Zn deficiency response and tolerance is still limited.

microRNAs (miRNAs) are endogenous small noncoding RNAs which are 20 to 24 nucleotides in size and generated from a single-stranded RNA precursor with a hairpin secondary structure [25]. They are known to direct target mRNA cleavage, translational repression, and DNA methylation on the basis of sequence complementarity with their target genes. In plants, miRNAs mainly direct mRNA cleavage and have important functions in the regulation of growth and development and various environmental stress responses [26–32]. For example, miR156 is involved in controlling rice grain size, panicle branching, crown root development and flowering time by targeting SPL transcription factor genes [27, 33, 34]; miR164 regulates lateral root development, leaf senescence, grain yield and drought stress tolerance [35–37]. miRNAs also regulate plant responses to multiple nutrient deprivation stresses and are involved in nutrient homeostasis regulation [38–40]. For instance, miR399, miR827 and miR778 are induced by phosphate deficiency and are involved in regulating phosphate homeostasis by regulating ubiquitin-conjugating E2 enzyme *PHO2*, RING-type ubiquitin E3 ligase *NLA*, and histone H3 lysine 9 (H3K9) methyltransferase *SUVH6*, respectively [40–43]; miR397, miR398, miR408 and miR857 are involved in Cu homeostasis regulation by controlling the expression of Cu-containing proteins [39, 44]. By using miRNA microarray, eight miRNA families were identified to be responsive to Zn deficiency in *Sorghum bicolor*, and two Cu/Zn superoxide dismutase genes *SbCSD1* and *SbCSD2* were found be targeted by miR398 and miR528, respectively [2]. However, to our knowledge, there is still no report on whether and how miRNAs regulate Zn deficiency responses in rice. In this study, we investigated the molecular mechanisms in response to Zn deficiency by integrating miRNAome and transcriptome analyses in rice seedlings. A comprehensive and integrated analysis of these different datasets have identified potential miRNA-mRNA interactions under Zn deficiency in rice.

## Results

### Transcriptome profilings in response to Zn deficiency and Zn resupply by RNA sequencing

**Fig. 1:**
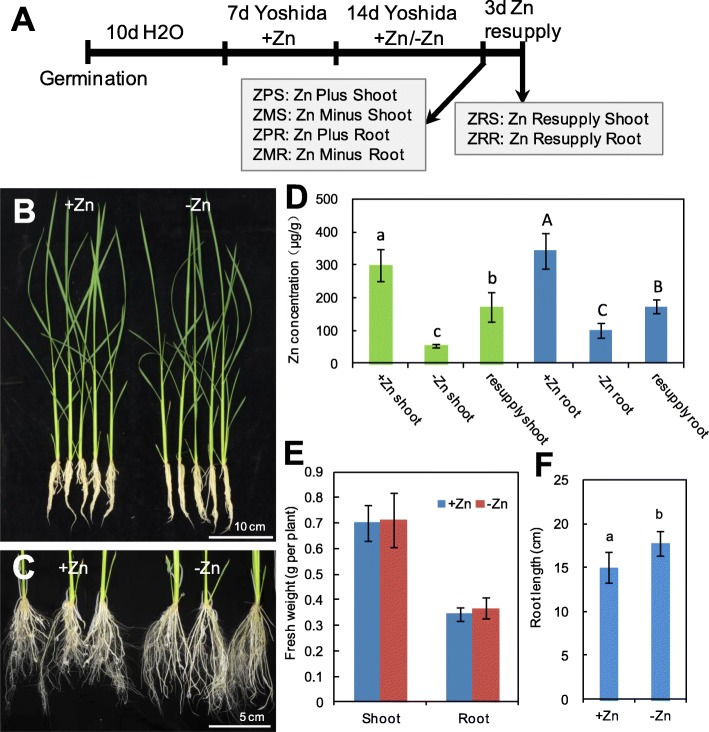
Physiological responses of rice seedlings to Zn deficiency and Zn resupply. **a** Schematic representation of the experimental design showing the duration of seedling growth and treatments of Zn deficiency and Zn resupply. Morphological appearance of the whole seedlings (**b**) and the roots (**c**) after Zn deficiency treatment for 14 days. **d** Zn concentration in the shoots and roots of rice seedlings after Zn deficiency for 14 days and Zn resupply for 3 days. Fresh weight of shoots and roots (**e**) and root length (**f**) of rice seedlings after Zn deficiency treatment for 14 days. Data are means ± SD from 3 samples for (**d**), and 10 samples for (**e** and **f**). Different letters indicate that the values are significantly different at *P* < 0.05 (Student’s t-test)

After experiencing Zn deficiency for 14 days, the Zn concentrations in shoots and roots were significantly decreased, and the Zn concentrations in shoots and roots of Zn-deficient seedlings were then significantly increased after Zn resupply for 3 days (Fig. 1a, b-d). No significant difference was observed in shoot and root biomass under Zn deficiency (Fig. 1e). Consistent with the phenotype observed in *Sorghum* [2], the primary root length were dramatically increased by Zn deficiency (Fig. 1c, f). These results showed that physiological adaptive responses to -Zn and Zn resupply have been exhibited.

We then analyzed the global transcriptome profiles of rice shoots and roots in responses to -Zn and Zn resupply by RNA sequencing. The Zn resupply treatments were used to investigate the recovery of Zn-responsive genes or miRNAs under Zn stress. By Illumina’s deep sequencing, a total of 28.6 to 55.2 million reliable clean reads were obtained from each library after excluding the low-quality reads, and most of the clean reads (86.8–97.3%) from each library could be mapped to the rice reference genome (https://rapdb.dna.affrc.go.jp/) (Additional file 1: Table S2). The Pearson’s correlation (R value) of the three biological replicates of each sample was around 90%, indicating the high reliability of the replicates (Additional file 2: Figure S1). The abundance of mapped transcripts was measured in terms of FPKM, and a total of 34,716 gene loci were detected in all these samples (Additional file 1: Table S3).

**Fig. 2:**
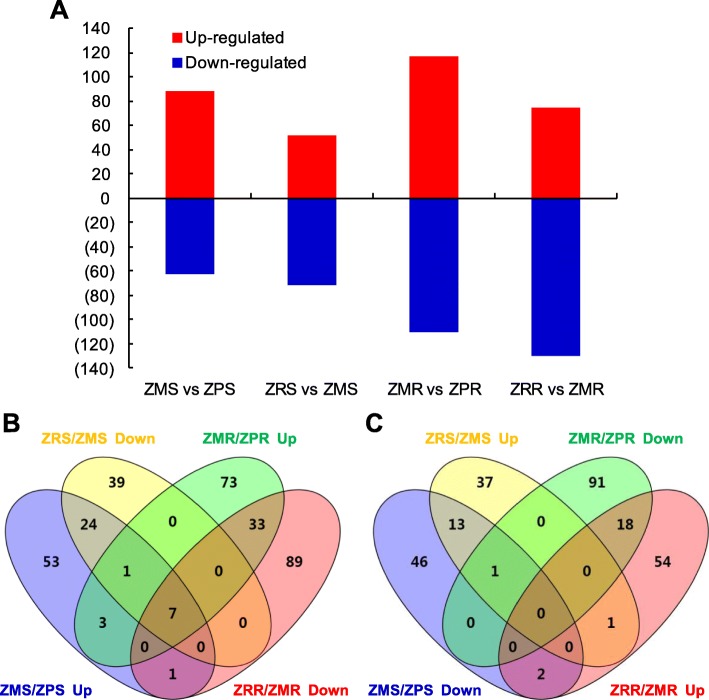
Overview of the differentially expressed genes (DEGs) in responses to 14 d of Zn deprivation and/or 3 d of Zn resupply. **a** The number of DEGs in responses to Zn deprivation and/or Zn resupply in rice roots and leaves (P < 0.05, FDR < 0.05). **b** Venn diagram representing the overlap of the Zn deficiency up-regulated DEGs and Zn resupply down-regulated DEGs in roots and leaves. **c** Venn diagram representing the overlap of the Zn deficiency down-regulated DEGs and Zn resupply up-regulated DEGs in roots and leaves

Differential expression analysis (fold change ≥2 and FDR ≤ 0.05) showed that a total of 151, 227, 123, and 205 genes were differentially expressed in Zn-deficient shoots (ZMS/ZPS), Zn-deficient roots (ZMR/ZPR), Zn-resupply shoots (ZRS/ZMS) and Zn-resupply roots (ZRR/ZMR), respectively (Fig. 2a; Additional file 1: Tables S4-S7). Venn diagram analysis showed that a total of 97 Zn-deficiency-responsive genes were recovered by Zn resupply in shoots and/or roots (Fig. 2b and c), suggesting a causal relationship between the expression levels of these genes and the levels of Zn in the growth media. Among the 89 up-regulated and 62 down-regulated genes in shoots (ZMS/ZPS up and down), 32 (36.0%) and 14 (22.6%) genes were recovered after Zn resupply treatment (ZRS/ZMS down and up), respectively (Table 1). Among the 117 up-regulated and 110 down-regulated genes in roots (ZMR/ZPR up and down), 40 (34.2%) and 18 (16.4%) genes were recovered after Zn resupply treatment (ZRR/ZMR down and up), respectively (Table 2). Seven genes (*Os10g0328600*, *Os04g0280500*, *Os04g0561500*, *Os06g0566201*, *Os06g0566300*, *Os08g0207500*, and *Os07g0232800*) were commonly recovered by Zn resupply in both roots and shoots; three of them encode Zn transporters (OsZIP4, OsZIP8 and OsZIP10). GO enrichment analysis (FDR < 0.05) revealed that zinc ion transmembrane transport (GO:0071577) and zinc ion transmembrane transporter activity (GO:0005385) were significantly enriched in Zn-deficiency up-regulated DEGs and Zn-resupply down-regulated DEGs in both shoots and roots, while threonine synthase activity (GO:0004795) and L-methionine biosynthetic process from methylthioadenosine (GO:0019509) were significantly enriched in Zn-deficiency down-regulated DEGs in shoots and roots, respectively (Additional file 1: Table S8).

**Table 1:** Zn deficiency-induced or repressed genes returning to basal level after Zn resupply in the shoots. The FPKM value represents mean ± SD (standard deviation) of three biological replicates. Note: MSTRG.18312, MSTRG.5686, and MSTRG.5688 are three new loci. “-” means no description

Gene	Description	ZPS (FPKM)	ZMS (FPKM)	ZRS (FPKM)
Os02g0192700	Similar to Thioredoxin peroxidase	0	68.55 ± 60.14	0
Os02g0663100	GRAS transcription factor domain containing protein	0	22.24 ± 4.50	0
Os05g0102000	SAM dependent carboxyl methyltransferase family protein	0	19.82 ± 17.40	0
Os10g0493300	Hypothetical conserved gene	0	13.02 ± 13.99	0
Os04g0382900	Conserved hypothetical protein	0	6.38 ± 9.55	0
Os01g0504100	Protein of unknown function DUF250 domain containing protein	0	6.10 ± 5.29	0
Os10g0528400	Glutathione S-transferase, C-terminal-like domain containing protein	0	5.39 ± 4.72	0
Os12g0103500	Ribosome-inactivating protein domain containing protein	0	4.59 ± 4.11	0
Os09g0116600	Hypothetical gene	0	4.06 ± 5.68	0
Os11g0490300	Hypothetical conserved gene	0	3.26 ± 2.85	0
Os03g0746700	Conserved hypothetical protein	0	3.08 ± 2.72	0
Os01g0532300	Conserved hypothetical protein	0	2.28 ± 2.08	0
Os06g0208951	Conserved hypothetical protein	0	2.00 ± 1.81	0
Os05g0562800	Similar to predicted protein	0	1.90 ± 2.55	0
Os03g0272900	DVL family protein	0	1.69 ± 1.59	0
Os09g0444800	Protein of unknown function DUF716 family protein	0	1.57 ± 1.36	0
Os10g0328600	–	0	1.39 ± 0.88	0
Os02g0550400	Tetratricopeptide-like helical domain containing protein	0	1.10 ± 0.95	0
Os07g0586900	GRAS transcription factor domain containing protein	0	1.02 ± 1.00	0
Os09g0412900	Pentatricopeptide repeat domain containing protein	0	0.64 ± 0.64	0
Os11g0606200	Similar to Leucine Rich Repeat family protein	0	0.49 ± 0.49	0
Os10g0328600	Hypothetical protein	0.02 ± 0.01	8.27 ± 1.81	0.18 ± 0.09
Os04g0280500	Similar to Non-S-locus F-box-like protein 2011	0.03 ± 0.03	10.03 ± 1.61	0.49 ± 0.22
Os04g0561500	Similar to Prolyl endopeptidase (Post-proline cleaving enzyme)	1.78 ± 0.63	204.72 ± 11.10	18.07 ± 8.12
Os06g0566201	–	1.38 ± 0.28	20.93 ± 10.37	1.48 ± 0.19
Os02g0306401	Similar to Nicotianamine aminotransferase A	5.81 ± 1.28	76.61 ± 8.44	15.04 ± 5.84
Os06g0566300	Zinc transporter OsZIP10	2.97 ± 0.26	38.94 ± 10.62	1.84 ± 0.34
MSTRG.18312	–	0.18 ± 0.02	1.92 ± 0.42	0.17 ± 0.08
Os08g0207500	Zinc transporter OsZIP4	1.7 ± 0.42	16.32 ± 8.27	1.9 ± 0.39
Os07g0232800	Zinc transporter OsZIP8	6.84 ± 1.31	50.73 ± 5.05	11.81 ± 6.25
Os03g0293100	Conserved hypothetical protein	7.25 ± 0.67	44.01 ± 14.47	7.78 ± 2.48
Os09g0485900	Similar to 60S ribosomal protein L9 (Gibberellin-regulated protein)	35.05 ± 6.96	164.62 ± 105.32	29.16 ± 13.21
Os11g0577866	Conserved hypothetical protein	9.27 ± 2.32	0	7.93 ± 4.44
Os06g0715200	Conserved hypothetical protein	8.48 ± 7.9	0	15.11 ± 6.49
Os08g0526100	NAD(P)-binding domain containing protein	6.15 ± 5.96	0	4.27 ± 3.76
Os01g0243450	Non-protein coding transcript	3.96 ± 0.68	0	1.66 ± 1.54
Os05g0528701	Non-protein coding transcript	1.81 ± 1.59	0	1.36 ± 1.27
Os01g0856800	Pleckstrin homology-type domain containing protein	0.86 ± 0.8	0	1.14 ± 0.98
Os09g0460300	Alpha/beta hydrolase fold-3 domain containing protein	0.8 ± 0.74	0	9.12 ± 8.68
Os08g0451201	–	0.69 ± 0.67	0	0.85 ± 0.76
Os03g0411900	–	0.64 ± 0.73	0	0.35 ± 0.32
Os01g0880250	Hypothetical protein	6.77 ± 6.57	0.03 ± 0.02	3.64 ± 5.01
MSTRG.5686	–	78.84 ± 109.73	0.42 ± 0.31	227.64 ± 346.33
Os06g0639800	Cytochrome P450 family protein	5.69 ± 0.85	0.06 ± 0.10	5.2 ± 2.26
MSTRG.5688	–	7.28 ± 9.58	0.20 ± 0.06	23.02 ± 34.31
Os03g0592500	–	66.95 ± 83.98	5.22 ± 1.37	30.1 ± 25.42

**Table 2:** Zn deficiency-induced or repressed genes returning to basal level after Zn resupply in the roots. The FPKM value represents mean ± SD of three biological replicates. Note: MSTRG.6046, MSTRG.22477, and MSTRG.30070 are three new loci. “-” means no description

Gene	Description	ZPR (FPKM)	ZMR (FPKM)	ZRR (FPKM)
Os08g0235800	Similar to WRKY transcription factor 25	0	23.49 ± 22.4	0
Os01g0896400	Similar to JHL23J11.5 protein	0	11.66 ± 18.26	0
Os01g0693800	Similar to Threonine synthase, chloroplast precursor	0	11.17 ± 9.72	0
Os05g0121500	Similar to structural constituent of ribosome	0	6.57 ± 6.92	0
Os03g0191900	Pathogenesis-related transcriptional factor and ERF domain containing protein	0	5.76 ± 5.66	0
Os11g0107600	Similar to prenylated Rab receptor 2	0	5.35 ± 4.77	0
Os02g0455400	Hypothetical conserved gene	0	4.66 ± 4.04	0
Os03g0852400	Conserved hypothetical protein	0	4.28 ± 3.71	0
Os03g0280750	Plant disease resistance response protein family protein	0	3.48 ± 3.11	0
Os05g0571600	Conserved hypothetical protein	0	3.2 ± 2.77	0
Os02g0310400	Conserved hypothetical protein	0	3.08 ± 2.68	0
Os05g0525900	Similar to Zing finger transcription factor PEI1	0	2.67 ± 2.32	0
Os01g0742300	3-hydroxyacid dehydrogenase/reductase domain containing protein	0	1.96 ± 1.79	0
Os03g0253200	Similar to WAG1; kinase	0	1.91 ± 1.66	0
Os06g0192800	Similar to RING-H2 finger protein ATL1R (RING-H2 finger protein ATL8)	0	1.59 ± 1.78	0
Os01g0234700	Harpin-induced 1 domain containing protein	0	1.25 ± 1.4	0
Os03g0103400	GRAS transcription factor domain containing protein	0	0.99 ± 0.89	0
Os10g0490100	Virulence factor, pectin lyase fold family protein	0	0.98 ± 0.88	0
Os03g0245700	Similar to senescence-associated protein 15	0	0.96 ± 0.84	0
Os05g0578100	Protein of unknown function DUF1645 family protein	0	0.82 ± 0.15	0
Os01g0584300	–	0	2.42 ± 2.23	0
Os04g0685700	Similar to H0723C07.4 protein	0	0.75 ± 0.65	0
Os07g0585900	Hypothetical conserved gene	0	0.75 ± 0.66	0
MSTRG.6046	–	0	0.66 ± 0.7	0
Os02g0242900	Similar to hydroquinone glucosyltransferase	0	0.63 ± 0.6	0
Os11g0256100	Pentatricopeptide repeat domain containing protein	0	0.41 ± 0.35	0
Os12g0137700	Sulfotransferase family protein	0.01 ± 0.01	3.9 ± 3.66	0.03 ± 0.04
Os04g0280500	Similar to Non-S-locus F-box-like protein 2011	0.07 ± 0.02	18.01 ± 5.28	0.31 ± 0.22
Os10g0553600	Exostosin-like family protein	0.01 ± 0.01	1.69 ± 0.89	0.01 ± 0.01
Os08g0207401	Hypothetical gene	0.01 ± 0.03	0.97 ± 0.34	0.04 ± 0.03
Os10g0328600	Hypothetical protein	0.11 ± 0.09	6.52 ± 1.13	0.13 ± 0.03
Os06g0566201	–	0.4 ± 0.03	13.82 ± 8.44	0.88 ± 0.15
Os06g0566300	Zinc transporter OsZIP10	0.88 ± 0.12	23.97 ± 7.22	1.58 ± 0.07
Os04g0561500	Similar to Prolyl endopeptidase (Post-proline cleaving enzyme)	7.38 ± 0.4	155.99 ± 10.59	10.99 ± 4.64
Os05g0472400	Similar to Zinc transporter 9	1.48 ± 0.25	29.79 ± 5.17	5.12 ± 2.05
Os07g0232800	Zinc transporter OsZIP8	0.75 ± 0.23	14.84 ± 1.15	0.99 ± 0.31
Os04g0304400	Similar to MADS-box protein AGL16-II	0.51 ± 0.04	8.26 ± 3.48	1.83 ± 0.09
Os08g0207500	Zinc transporter OsZIP4	2.9 ± 0.24	28.12 ± 9.16	4.29 ± 1.91
MSTRG.22477	–	0.34 ± 0.11	3.12 ± 0.97	0.09 ± 0.06
MSTRG.30070	–	21.6 ± 19.8	91.8 ± 29.43	0.46 ± 0.24
Os06g0131300	Arginine decarboxylase, Chilling stress respons	98.89 ± 73.37	0	123.68 ± 112.96
Os03g0244950	Heat shock protein DnaJ, N-terminal domain containing protein	6.6 ± 7.68	0	3.45 ± 3.04
Os06g0505501	Non-protein coding transcript	3.46 ± 1.52	0	1.61 ± 1.6
Os02g0756850	Hypothetical protein	2.36 ± 0.31	0	1.33 ± 1.33
Os11g0706200	GRAS transcription factor domain containing protein	1.8 ± 1.63	0	1.98 ± 1.91
Os12g0124700	CDC45-like protein family protein	1.16 ± 1.08	0	1.83 ± 2.03
Os09g0412900	Pentatricopeptide repeat domain containing protein	1.12 ± 0.98	0	0.96 ± 0.84
Os03g0140300	Conserved hypothetical protein	0.99 ± 1.07	0	0.62 ± 0.54
Os04g0277400	Membrane bound O-acyl transferase, MBOAT family protein	0.84 ± 0.71	0	1.14 ± 1.09
Os10g0328700	Tetratricopeptide-like helical domain containing protein	0.58 ± 0.47	0	0.39 ± 0.34
Os07g0172600	Pentatricopeptide repeat domain containing protein	0.55 ± 0.58	0	0.67 ± 0.63
Os01g0163000	Leucine-rich repeat, N-terminal domain containing protein	0.46 ± 0.1	0	0.4 ± 0.38
Os03g0821700	Pentatricopeptide repeat domain containing protein	0.45 ± 0.15	0	0.53 ± 0.19
Os02g0621600	Similar to OSIGBa0157K09-H0214G12.24 protein	0.44 ± 0.59	0	0.62 ± 0.4
Os11g0133500	Serine/threonine protein kinase-related domain containing protein	0.35 ± 0.31	0	0.71 ± 0.8
Os12g0585300	Cyclin-like F-box domain containing protein	1.17 ± 1	0	1.53 ± 1.33
Os11g0111000	Conserved hypothetical protein	1.1 ± 0.92	0.01 ± 0.01	1.07 ± 1.11
Os03g0758500	Similar to blue copper protein	6.31 ± 5.39	0.15 ± 0.1	11.47 ± 3.69

### miRNA profilings in rice responses to Zn deficiency and Zn resupply

Sequencing results showed that 8.8 to 15.3 million raw reads were obtained for each of the 18 libraries, and after discarding low-quality, junk and adaptor reads, repeats, and Rfam RNA and mRNA sequences, 38.3 to 56.0% of the total reads were valid reads in these libraries (Additional file 1: Table S9). The size distribution analysis showed that small RNAs from the six samples were primarily enriched in sequences with 21- to 24-nt, and 24-nt was the most dominant segment (Additional file 1: Table S10; Additional file 2: Figure S2), which is in concurrence with previous studies [45]. Pearson correlation analysis of three biological replicates of each sample revealed high correlation coefficients ranging from 0.80 to 0.99 (Additional file 2: Figure S3), indicating the high reliability of the replicates. The clean reads were searched against the miRNAs of rice and other plant species from miRBase, and a total of 498 known/conserved miRNAs were identified from all samples. The reads which could not be mapped to miRBase were subjected to novel miRNA prediction. After the removal of small RNAs which do not meet the plant miRNA criteria (eg. length < 20 or > 24, no hairpin structure, less than 10 reads in a sample), a final set of 370 novel miRNA genes was obtained (Additional file 1: Table S11).

**Fig. 3:**
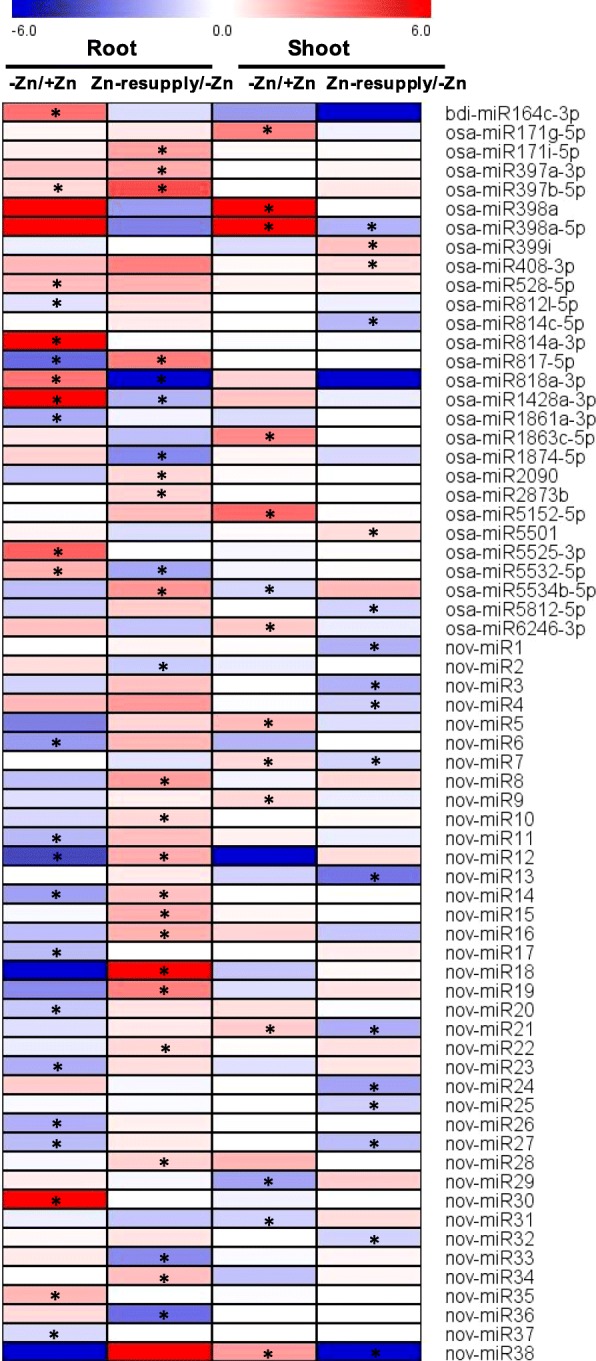
Heatmap representation for expression profiles of miRNAs in response to 14 d of Zn deprivation and/or 3 d of Zn resupply in the roots and shoots. The intensities of the color represent the relative magnitude of fold changes in log2 values according to small RNA high-throughput sequencing data. The asterisks indicate significantly differentially expressed miRNAs with an absolute fold change ≥2 and *P*-value ≤0.05. Red color indicates induction, blue color indicates repression.

A total of 68 miRNAs (including 38 novel miRNAs) were found to be differentially expressed under Zn deficiency (ZMS/ZPS in shoots and ZMR/ZPR in roots) and/or Zn resupply (ZRS/ZMS in shoots and ZRR/ZMR in roots); 38 miRNAs were differentially expressed under Zn deficiency and 44 miRNAs were differentially expressed under Zn resupply; 14 miRNAs were commonly responsive to Zn deficiency and Zn resupply (Additional file 1: Table S12; Fig. 3; Additional file 2: Figures S4 and S5). Of these Zn-responsive miRNAs, 12 and 11 were up-regulated by Zn deficiency in shoots and roots, respectively; 2 and 12 were down-regulated by Zn deficiency in shoots and roots, respectively (Additional file 2: Figure S4). However, none of these miRNAs were commonly responsive to Zn deficiency in both shoots and roots, suggesting the distinct miRNA-mediated regulatory networks in shoots and roots under Zn deficiency. Seventeen and 27 miRNAs were found to be responsive to Zn resupply in shoots and roots, respectively. Interestingly, four and seven Zn-deficiency-responsive miRNAs could be recovered by Zn resupply in shoots and roots, respectively (Additional file 2: Figure S4). For example, osa-miR398a-5p, nov-miR7, nov-miR21, and nov-miR38 were induced by Zn deficiency but were repressed by Zn resupply in shoots; osa-miR818a-3p, osa-miR1428a-3p, and osa-miR5532-5p were induced by Zn deficiency but were repressed by Zn resupply in roots (Fig. 3). Two miRNAs showed similar expression pattern under Zn deficiency and Zn resupply; miR397b-5p was induced by both Zn deficiency and Zn resupply in roots, and nov-miR27 was repressed by Zn deficiency and Zn resupply in roots and shoots, respectively (Fig. 3), suggesting the complex regulations of these miRNAs under Zn deficiency and resupply.

### Identification of potential target genes of Zn-responsive miRNAs

To understand the potential biological function of these Zn-deficiency- and/or Zn-resupply-responsive miRNAs, miRNA-targeted genes were predicted using an online tool psRNATarget [46]. A total of 799 potential target genes were identified for these 68 Zn-responsive miRNAs (Additional file 1: Table S13). At least 19 target genes of the four Zn-responsive miRNAs (miR398, miR408, miR528, miR397) have been previously validated through experiments like RNA ligase-mediated 5′ rapid amplification of cDNA ends (RLM 5′-RACE), degradome sequencing, and genetic analysis (Table 3) [47–49]; most of them were Cu-containing proteins and were involved in Cu homeostasis, reactive oxygen species (ROS) homeostasis, photosynthesis and stress tolerance [49–58].

**Table 3:** The validated target genes of some Zn-responsive miRNAs

miRNA	Target gene	Target gene annotation	Potential function
osa-miR398a	Os07g0665200	Cu/Zn-superoxidase dismutase2/CSD2	Stress tolerance, antioxidant defense, copper homeostasis
	Os03g0351500	Cu/Zn-superoxidase dismutase1/CSD1	
	Os11g0203300	Superoxide dismutaseX/SODX	
	Os04g0573200	Copper chaperone for superoxide dismutase/CCSD	
osa-miR408-3p	Os08g0482700	Uclacyanin-like protein UCL30	Stress tolerance, photosynthesis, copper homeostasis,
	Os03g0709100	Uclacyanin-like protein UCL8	
	Os04g0545600	Uclacyanin-like protein UCL15	
	Os06g0218600	Uclacyanin-like protein UCL16	
	Os02g0653200	Plantacyanin like 2/Uclacyanin-like protein UCL4	
	Os02g0731400	Plantacyanin-like 3/Uclacyanin-like protein UCL5	
	Os02g0758800	Plantacyanin-like 4/Uclacyanin-like protein UCL6	
	Os01g0827300	Laccase 3/LAC3	
	Os03g0297900	Laccase 12/LAC12	
osa-miR528-5p	Os06g0567900	Ascorbate oxidase 2	Reactive oxygen species homeostasis, stress tolerance, copper homeostasis, strigolactone signal perception
	Os08g0137400	Uclacyanin-like protein UCL23	
	Os07g0570550	Cupredoxin domain containing protein	
	Os06g0154200	F-box/LRR-repeat MAX2 homolog/DWARF3	
	Os08g0561700	Superoxide dismutase 4/CSD4	
osa-miR397b-5p	Os05g0458600	Laccase 15/LAC15	Copper homeostasis, lignin biosynthesis, brassinosteroid response

**Fig. 4:**
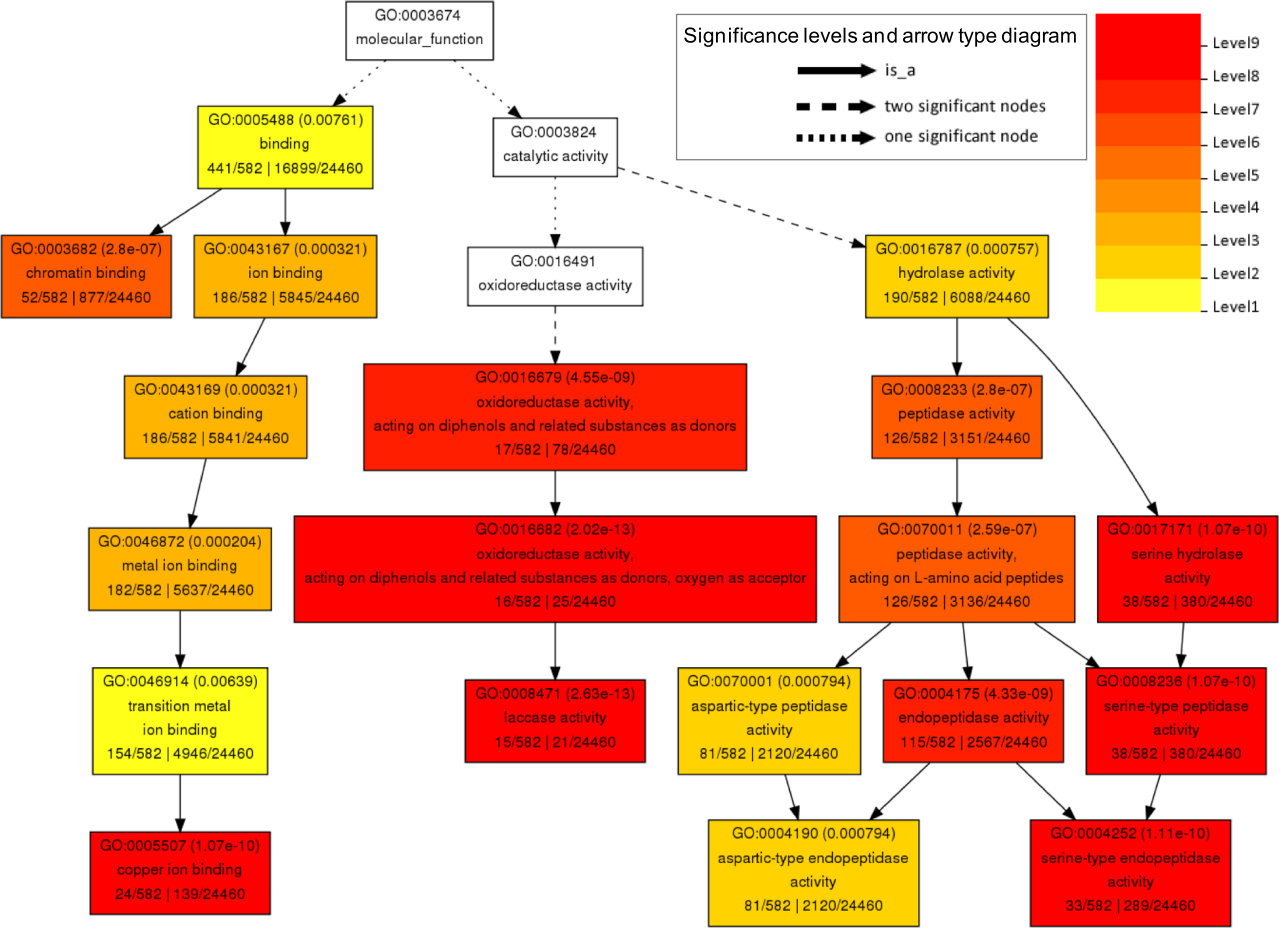
Gene ontology (GO) representation of the overrepresented GO terms of molecular functions in the potential target genes of the differentially expressed miRNAs under Zn deprivation and/or Zn resupply. The GO representation was generated using single enrichment analysis (SEA) tool on AgriGO (http://bioinfo.cau.edu.cn/agriGO/) (Fisher’s test, P < 0.05, FDR < 0.05). The number in parenthesis represents the FDR value

To further elucidate the potential biological functions of the predicted target genes in Zn deficiency response, GO enrichment analysis was performed. In total, 26 GO terms of biological process group, 19 GO terms of molecular function group, and 18 GO terms of cellular component group were significantly enriched (FDR < 0.05) ( Fig. 4; Additional file 2: Figure S6 and S7). Among the enriched molecular functions, the most significant GO terms were oxidoreductase activity (GO:0016682), laccase activity (GO:0008471), serine-type peptidase activity (GO:0008236), Cu ion binding (GO:0005507), and serine hydrolase activity (GO:0017171) (Fig. 4). Among the enriched biological processes, the most enriched GO terms were lignin metabolic process (GO:0009808), phenylpropanoid catabolic process (GO:0009808), cellular amino acid derivative catabolic process (GO:0042219), secondary metabolic process (GO:0019748), and proteolysis (GO:0006508) (Additional file 2: Figure S6). In cellular component group, apoplast (GO:0048046), chromatin (GO:0000785), and photosynthetic membrane (GO:0034357) were the most over-represented terms (Additional file 2: Figure S7).

### Integrated analysis of miRNAome and transcriptome

Given that miRNAs negatively regulate the expression of their target mRNAs by target cleavage, the expression patterns of miRNAs generally show an inverse correlation with those of their target genes. By integrating transcriptome and miRNAome data, twelve DEGs under -Zn and/or Zn resupply were predicted to be targeted by 10 miRNAs (including four known and six novel miRNAs) that were responsive to -Zn and/or Zn resupply (Table 4). Half of the potential target DEGs showed a different response to Zn deficiency or Zn resupply as compared with the corresponding miRNAs, suggesting the negative regulation of these miRNAs on their potential target genes. For example, miR397b-5p was up-regulated by Zn resupply, while its potential target genes *OsLAC11* (Os03g0273200) and *OsLAC29* (Os12g0258700) were up-regulated by -Zn or down-regulated by Zn resupply; osa-miR398a-5p was up-regulated by Zn deficiency, while its potential target gene *Os07g0213800* was down-regulated; nov-miR4 was down-regulated by Zn resupply, while its potential target gene *Os01g0358700* was up-regulated; nov-miR6 was down-regulated by Zn deficiency, while it potential target gene *Os04g0623901* was up-regulated; nov-miR23 was down-regulated by Zn deficiency, while its potential target gene *Os01g0923900* was down-regulated by Zn-resupply. However, half of the potential target genes showed similar expression patterns with the miRNAs (Table 4). This may be caused by the following reasons: these genes are inauthentic targets of the miRNAs; there are complicated regulation of the potential target genes; the potential target genes are regulated by translational repression but not by transcript cleavage.

**Table 4:** DEGs under Zn deficiency and/or Zn resupply that are potential targets of Zn-responsive miRNAs

miRNA	miRNA response to Zn	Potential target	Target description	Target response to Zn
osa-miR171g-5p	-Zn up	Os05g0278500	Transferase family protein	-Zn up
osa-miR397b-5p	Zn resupply up	Os03g0273200	LACCASE 11	-Zn up
		Os12g0258700	LACCASE 29	Zn resupply down
osa-miR398a-5p	-Zn up, Zn resupply down	Os07g0213800	Similar to Allergenic protein	-Zn down
osa-miR528-5p	-Zn up	Os06g0154200	Similar to F-box/LRR-repeat MAX2 homolog	-Zn up
nov-miR1	Zn resupply down	Os08g0107800	Pentatricopeptide repeat domain containing protein	Zn resupply down
nov-miR4	Zn resupply down	Os01g0358700	–	Zn resupply up
nov-miR6	-Zn down	Os04g0623901	Non-protein coding transcript	-Zn up
nov-miR23	-Zn down	Os06g0505501	Non-protein coding transcript	-Zn down, Zn resupply up
		Os01g0923900	Cyclin-like F-box domain containing protein	Zn resupply down
nov-miR26	-Zn down	Os11g0684700	Similar to NB-ARC domain containing protein	Zn resupply up
nov-miR38	-Zn up, Zn resupply down	Os04g0304400	Similar to MADS-box protein AGL16-II	-Zn up, Zn resupply down

### Confirmation of miRNAome and transcriptome results by qRT-PCR

**Fig. 5:**
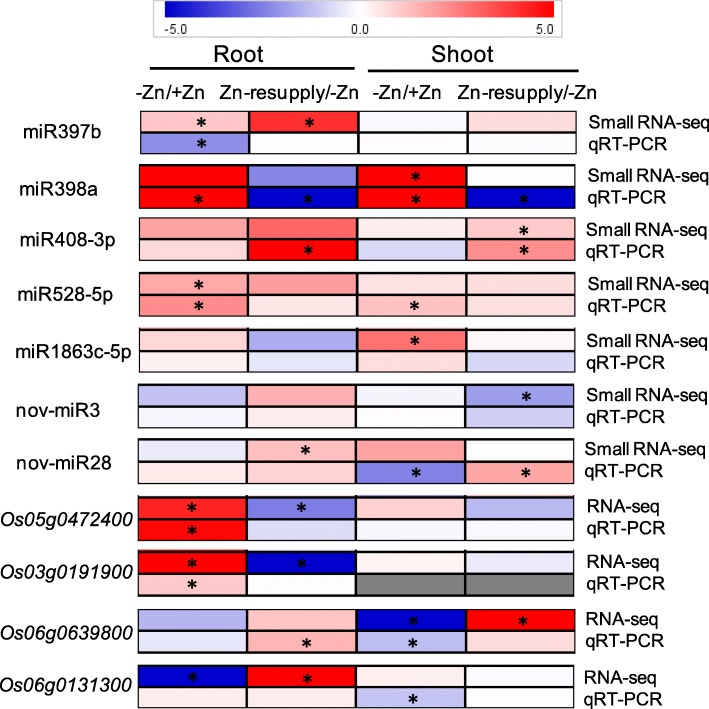
Heatmap of the expression of differentially expressed miRNAs and DEGs revealed by deep sequencing and qRT-PCR. Seven miRNAs and four DEGs were randomly selected and analyzed by qRT-PCR. The primary transcripts of miRNAs (pri-miRNAs) were analyzed to for miRNA expression validation. The intensities of the color represent the fold changes in log2 values according to qRT-PCR results of three replicates. The asterisks indicate an absolute fold change ≥2 and P-value ≤0.05. Red color indicates induction, blue color indicates repression, and gray color indicates no expression detected

To confirm the small RNA sequencing results, seven Zn-responsive miRNAs were randomly selected for expression validation by qRT-PCR. Primary miRNAs (pri-miRNAs), which contain a characteristic hairpin structure, are transcribed from MIR genes through polymerase II in plants and can be used to indicate the expression level of mature miRNAs [59]. Here, the primary transcripts of these miRNAs were analyzed using gene-specific primers based on the sequences of the corresponding miRNA genes. The expression of pri-miRNAs were almost consistent with the small RNA sequencing results of mature miRNAs. For example, pri-miR398a was up-regulated by Zn deficiency and down-regulated by Zn resupply; pri-miR408 was up-regulated by Zn resupply; pri-miR528 was up-regulated by Zn deficiency (Fig. 5). However, pri-miR397b and pri-nov-miR28 were repressed by Zn deficiency, which is inconsistent with the expression of mature miR397b and nov-miR28, suggesting the different responses of these pri-miRNAs and mature miRNAs under Zn deficiency. To confirm the RNA sequencing results, four Zn-responsive genes (*Os05g0472400*, *Os03g0191900*, *Os06g0639800*, and *Os06g0131300*) were randomly selected and investigated by qRT-PCR. The expression patterns of these genes under Zn deficiency and recovery were similar to the results of RNA-seq, with the exception of *Os06g0131300* (Fig. 5). Significant expression changes of *Os06g0131300* in roots under Zn deficiency and recovery could not be observed by qRT-PCR, but its expression was found to be repressed by Zn deficiency in shoots as observed by qRT-PCR. The expression levels of *Os03g0191900* in shoots could not be detected by qRT-PCR, which is consistent with the low FPKM value in shoots as revealed by RNA-seq (Fig. 5). These results suggest that the data of small RNA sequencing and mRNA sequencing obtained in this study is reliable.

**Fig. 6:**
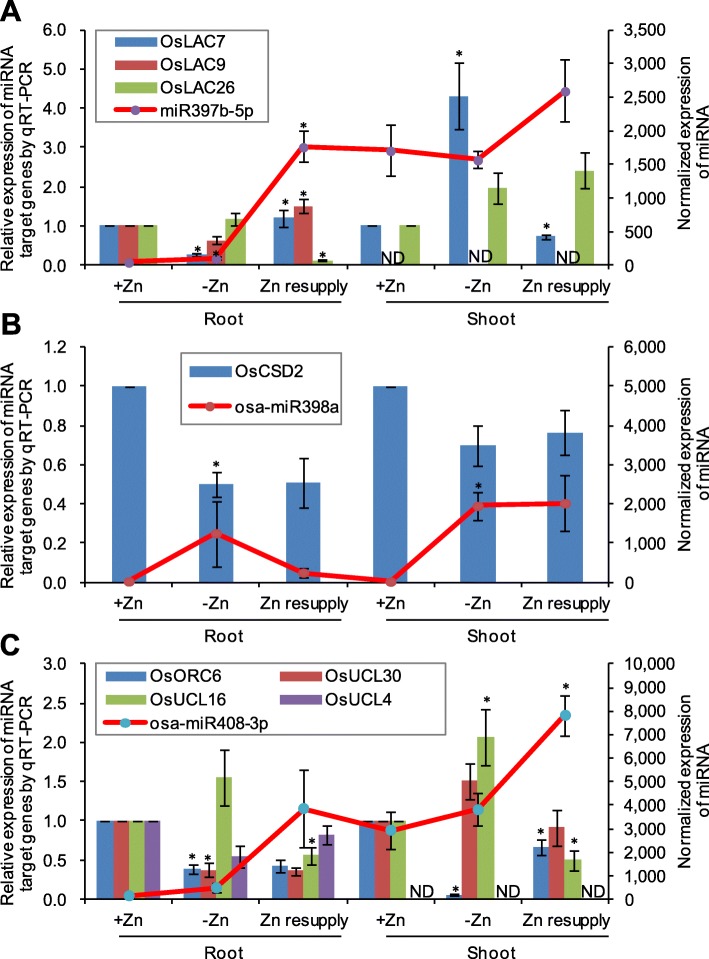
The relative expression levels of three Zn-responsive miRNAs and their potential target genes. The relative expression of potential target genes of miR397b-5p (**a**), miR398a (**b**), and miR408-3p (**c**) were analyzed by qRT-PCR. The normalized expression levels of miRNAs revealed by small RNA sequencing were shown with line charts. The expression level of +Zn control in roots and shoots was normalized to one. Error bar represents standard deviation of three biological replicates. The asterisk above error bar indicates an absolute fold change ≥2 (−Zn/+Zn, or Zn resupply/−Zn) and P-value ≤0.05. ND means “not detected”

We also analyzed the expression of several potential target genes of Zn-responsive miRNAs by qRT-PCR. The potential target genes of miR397b-5p showed differential responses to Zn deficiency; *OsLAC7* (Os01g0850550) and *OsLAC9* (Os01g0850800) were repressed by Zn deficiency in roots, which is contrary to the expression of miR397b-5p; *OsLAC7* was induced by Zn deficiency and repressed by Zn resupply in shoots (Fig. 6a). *OsCDS2*, the potential target gene of miR398a, was found to be repressed by Zn deficiency in roots, which is contrary to the expression of miR398a (Fig. 6b). The potential targets of miR408-3p also showed differential responses to Zn deficiency and Zn resupply, suggesting their different functions in Zn deficiency response. *OsUCL16* (Os06g0218600) was up-regulated by Zn deficiency and down-regulated by Zn resupply; *OsUCL30* (Os08g0482700) was down-regulated by Zn deficiency in roots; *OsORC6* (Os07g0628600) was down-regulated by Zn deficiency in both roots and shoots (Fig. 6c).

## Discussion

### Identification of DEGs in response to Zn deficiency and recovery

In this study, a total of 569 loci were found to be responsive to Zn deficiency and/or Zn resupply; 360 of them were responsive to Zn deficiency in roots and/or shoots, and 316 of them were responsive to Zn resupply in roots and/or shoots (Fig. 2). By comparing with earlier studies [24], 49 of the DEGs were reported to be responsive to Zn deficiency and/or Zn resupply (Additional file 1: Table S14). Five of the common DEGs are putative Zn transporters, and three of the common DEGs are metallothionein-like proteins, which are implicated in Zn homeostasis [7, 60]. In addition, 97 DEGs were found to be recovered by Zn resupply treatment in shoots and/or roots (Tables 1 and 2), suggesting their specific responses to Zn deficiency. These DEGs were enriched in GO terms of cellular metabolic process, primary metabolic process and biosynthetic process (Additional file 2: Figure S8), suggesting that these processes are affected by Zn deficiency and can be recovered by Zn resupply. Four of the DEGs (*Os03g0108300*, *Os10g0528400*, *Os12g0570700*, *Os12g0571000*) were also reported to be recovered by Zn resupply [24].

Twelve DEGs were predicted to be targeted by 10 Zn-responsive miRNAs, and half of them showed a differential expression with miRNAs under Zn deficiency and/or Zn resupply (Table 4), suggesting the interactions between miRNAs and their potential target genes. However, this cannot exclude the responsiveness of other potential target genes of Zn-responsive miRNAs. As revealed by qRT-PCR, several potential target genes of miR397b, miR408 and miR398a were also found to be induced or repressed by Zn deficiency and/or Zn resupply (Table 3, Fig. 5b). The number of DEGs potentially regulated by Zn-responsive miRNAs in this study is far below our expectation. One of the possible reasons is that the accepted standard used for the definition of DEGs may miss some interactions between miRNAs and their potential target genes, and more interaction pairs may be identified by lowering the threshold.

### miRNAs potentially involved in the regulation of Zn deficiency response in rice

miRNAs have been demonstrated to be involved in signaling and regulation of nutrient stress responses, like nitrogen deficiency, phosphate starvation, sulfate deprivation, and Cu deficiency [38–40, 61, 62]. In this study, we analyzed the miRNA expression profilings under Zn deficiency and Zn resupply, and 68 miRNAs were found to be responsive to Zn deficiency and/or Zn resupply in rice (Fig. 3). Some of the miRNA families were also found to be responsive to Zn deficiency in *Sorghum bicolor*, like miR171, miR398, miR399, miR408, and miR528 [2], suggesting their conserved roles in plant response to Zn deficiency.

Among the Zn-responsive miRNAs, 38 (56%) of them were novel miRNAs. Most of these novel miRNAs (36/38) are encoded by a single locus (Additional file 1: Table S12), suggesting their recent evolutionary origin. Most of the novel miRNAs (30/38) contained an opposite strand (miRNA*) that can be detected by small RNA sequencing, and most of the novel miRNAs (28/38) were 24 nt in length (Additional file 1: Table S12; Additional file 2: Figure S5). In rice, a proportion of pri-miRNAs can be processed into canonical miRNAs (21 nt) and long miRNAs (24 nt) by Dicer-like 1 (DCL1) and DCL3, respectively [63, 64]. The 24 nt long miRNAs are sorted into the effector Argonaute 4 (AGO4) and possibly direct DNA methylation by base pairing with target loci-derived transcripts [26, 63]. DNA methylation modulates gene expression and represses the transcription and movement of transposable elements. The expression of some key nutrient stress-responsive genes has been suggested to be regulated by DNA methylation under specific nutrient stress conditions [65]. In this study, many of the Zn-responsive novel miRNAs (20/38) were down-regulated by Zn deficiency and/or up-regulated by Zn resupply (Fig. 3). The potential target genes of these Zn-responsive novel miRNAs encode various kinds of proteins, such as transcription factors, transporters, protein kinases, Zn-containing protein, and oxidoreductases (Additional file 1: Table S13). Whether these recently-evolved and possibly species-specific Zn-responsive novel miRNAs are associated with acclimation to submergence and low Zn conditions in rice, and are involved in epigenetic regulation of Zn deficiency response deserve further investigations.

### Potential role of miRNAs in Cu-Zn interaction in plants

Among the Zn-responsive miRNAs identified in this study, some of the miRNA families were previously documented to be responsive to Cu deficiency, such as miR397, miR398, and miR408, which are all induced by Cu deficiency and their target genes encode various Cu-containing proteins [39]. In rice, nineteen potential target genes of the Cu/Zn-responsive miRNAs have been validated, most of them encode Cu-containing proteins, like CSDs, laccases, plantacyanin like proteins, and uclacyanin-like proteins (Table 3). The potential target genes of Zn-responsive miRNAs were significantly enriched in the GO terms of Cu ion binding and laccase activity (Fig. 4). These results suggest the involvement of miRNAs in the interaction between Cu and Zn nutrition in plants.

It has been demonstrated that Cu and Zn could interact in several ways: Cu competitively inhibits Zn absorption, Zn strongly depresses Cu absorption, and Cu affects the redistribution of Zn within plants [66, 67]. The uptakes of Cu and iron (Fe) were found to be increased by Zn deficiency in rice seedlings [68]. In this study, miR398 and miR528 were found to be induced by Zn deficiency. The induction of miR398 and miR528 could depress the expressions of Zn-containing target gene *CSD*s [2, 50], which can be functionally replaced by Fe superoxide dismutase and Mn superoxide dismutase, thus further allow the allocation of limited Zn to other essential Zn-containing proteins in order to adapt to the low Zn environment. The miRNA-mediated regulation of Zn homeostasis could be similar to that of the miRNA-mediated regulation of Cu homeostasis as reported previously [69, 70]. Interestingly, another two Cu-responsive miRNAs, miR397 and miR408 were found to be induced by Zn resupply (Fig. 3). The induction of these two miRNAs may be a direct effect of Zn resupply, and may also be an indirect effect of Zn-resupply-induced antagonism on Cu nutrition. The primary transcripts of miR397b and miR408 were found to be repressed by Zn deficiency (Fig. 5). Whether the repressions of miR397b and miR408 would be associated with the Zn deficiency-induced Cu accumulation in plant tissues deserve further studies.

### miRNA-mediated oxidative stress in response to Zn deficiency

Zn deficiency can interfere with membrane-bound NADPH oxidase producing ROS and further cause oxidative damage to critical cell compounds by the excessive ROS, which is a major factor affecting plant growth under Zn deficiency [11]. It has been reported that Zn deficiency causes cellular damage in leaves and roots and maintaining a higher ROS defense level is associated with reduced cellular damage in rice leaves [2, 71, 72]. In this study, GO terms of oxidoreductase activity was significantly enriched in the potential target genes of Zn-responsive miRNAs (Fig. 4). miR398 was reported to be involved in the regulation of oxidative stress response by silencing the expression of *CSD1* and *CSD2* in Arabidopsis [73]. Here, miR398 was induced by Zn deficiency in the shoots. The induction of miR398 could repress the expression of its target genes (*CSDs*, *SODX*, *CCSD*) and then promote the accumulation of ROS. miR398 was also reported to be induced by other stresses, such as salt, heat and infection of blast fungus *Magnaporthe oryzae* [29, 52]. The induction of miR398 has been suggested to be required for thermotolerance in Arabidopsis and resistance to blast disease in rice [51, 52]. Another Zn-responsive miRNA, miR528 was also reported to be involved in the regulation of ROS accumulation [55, 56]. One of the target gene of miR528, *CSD4* (*Os08g0561700*) is possibly involved in ROS scavenging. Another target gene of miR528, *Os06g0567900*, encoding a L-ascorbate oxidase (AO)*,* is involved in producing ROS by regulating the redox state of the apoplast by oxidizing apoplastic L-ascorbate acid [56]. Overexpression of osa-miR528 can decrease AO activity and increase ROS scavenging and further improve plant tolerance to salt and nitrogen deficiency stresses in creeping bentgrass [55]. In this study, miR528 was found to be induced by Zn deficiency, suggesting its role in balancing ROS accumulation and scavenging under Zn stress.

## Conclusions

The present study is the first attempt to integrate global miRNA and mRNA expression profiles to identify regulatory miRNA/target modules in response to Zn deficiency and recovery in rice. A set of 68 miRNAs including 38 novel miRNAs and five Cu-responsive miRNAs were identified to be differentially expressed. At least 12 potential target genes of Zn-responsive miRNAs were found to be altered by Zn deficiency and recovery. Ninety-seven Zn-deficiency-responsive genes were found to be recovered by Zn resupply. This global analysis of the rice miRNAome and transcriptome in responses to Zn deficiency and recovery identifies candidate miRNAs and genes that can be further characterized to increase our understanding of the molecular mechanisms of Zn deficiency response. The identified differentially expressed miRNA/target modules and genes can also be considered as candidates for improving Zn deficiency tolerance by genetic engineering approaches.

## Methods

### Plant material and growth conditions

Seeds of the rice (*Oryza sativa* L. ssp. *japonica cv. Nipponbare*) from our own lab were surface sterilized in 10% (v/v) H_2_O_2_ for 20 min and rinsed with sterile water [74]. Seeds were then transferred to seedling trays floating on sterile water for germination. After growing in the sterile water for 10 days, seedlings were transferred to a plastic container with 2.5 L Yoshida solution [75]. After growing in the nutrient solution (with 1.5 μM ZnSO_4_) for 1 week, uniform seedlings were used for Zn deficiency (−Zn) treatment. For -Zn treatment, no ZnSO_4_ was added to the nutrient solution. The fresh nutrient solution was replenished every 2 day, and the pH was adjusted to 5.6. Plants were grown in a growth chamber under controlled conditions. The light intensity was approximately 180 μmol m^− 2^ s^− 1^ at shoot height with a day/night cycle of 16 h/8 h at 26 °C/24 °C. The relative humidity was 50%. Roots and shoots of rice seedlings were collected after Zn-deficient treatment for 2 weeks. For Zn-resupply treatment, Zn-deficient seedlings were transferred to normal nutrient solution containing 1.5 μM ZnSO_4_ for 3 days. After Zn-resupply treatment, roots and shoots were collected for analyses at the same time point as collecting the Zn-deficient samples (4 hours after illumination).

### Zn content determination

Zn content was analyzed as described previously [23, 76]. Plant tissues were collected and dried in an oven at 80 °C for 3 days. The roots were rinsed with deionized water for three times before collection. The dried roots or shoots were ground, and about 0.10 g sample was used for analysis. After digestion with HNO_3_/HClO_4_ (4:1, v/v) at 180 °C for 4 h, the solution was diluted five times with 1% HNO_3_, and then the concentration of Zn was determined by Inductively Coupled Plasma Mass Spectroscopy (Perkin-Elmer NexION 300X, Waltham, MA).

### cDNA library construction and RNA sequencing

Rice shoots and roots were collected after Zn-deficiency and Zn-resupply treatments. Three rice seedlings were collected and mixed together as one sample. Eighteen samples (Zn plus shoot, ZPS; Zn minus shoot, ZMS; Zn plus root, ZPR; Zn minus root, ZMR; Zn resupply shoot, ZRS; Zn resupply root, ZRR, each with three biological replicates) were harvested for RNA library construction and sequencing. Total RNA was extracted using Trizol reagent (Invitrogen, CA, USA), and the quantity and quality were analyzed by Bioanalyzer 2100 and RNA 6000 Nano LabChip Kit (Agilent, CA, USA). The RIN value (RNA integrity number) of all RNA samples were more than 8.0. Ten μg of total RNA of each sample was subjected to isolate Poly (A) mRNA with poly-T oligo-attached magnetic beads (Invitrogen, Carlsbad, CA). After purification, the mRNA is fragmented into small pieces using divalent cations under elevated temperature. The cleaved RNA fragments were reverse-transcribed to create cDNA libraries according to the instructions of the mRNA-Seq sample preparation kit (Illumina, San Diego, USA). The average insert size of the paired-end libraries was 300 ± 50 bp. The paired-end sequencing was performed on an Illumina Hiseq4000 at the LC-BIO (Hangzhou, China). The RNA-seq datasets of this study were deposited in NCBI’s Gene Expression Omnibus and are accessible through GEO accession number GSE130980 (https://www.ncbi.nlm.nih.gov/geo/query/acc.cgi?acc=GSE130980).

### Mapping of RNA-seq reads and identification of differentially expressed genes

The initial base calling and quality filtering of the reads generated with the Illumina analysis pipeline (Fastq format) were conducted using a custom Perl script and the default parameters of the Illumina pipeline (http://www.illumina.com). Additional filtering for poor-quality bases was performed using the FASTX-toolkit available in the FastQC software package (http://www.bioinformatics.babraham.ac.uk/projects/fastqc/). The rice reference genome (version IRGSP-1.0) (https://rapdb.dna.affrc.go.jp/) was indexed by Bowtie2 to facilitate the read mapping. The read mapping was performed using the Tophat software (version 2.1.1) package [77]. Tophat allows multiple alignments per read (up to 40) and a maximum of two mismatches when mapping the reads to the reference genome. Reads were first mapped directly to the genome using indexing and the unmapped reads were used to identify novel splicing events. The aligned read files were processed by Cufflinks to measure the relative abundances of the transcripts by using the normalized RNA-seq fragment counts. The estimated gene abundance was measured in terms of the fragments per kilobase of transcript per million mapped reads (FPKM). The DEGs between the two treatments were identified using Cuffdiff (version 2.1.1). Only the genes with a log2 fold change ≥1 or ≤ − 1, and an adjusted *P*-value (FDR ≤ 0.05) were considered as significantly DEGs. The Pearson correlation was calculated and plotted using the R Stats package (version 3.5.0) based on the normalized counts of mRNAs of each sample.

### Functional annotation and gene ontology (GO) enrichment

Zn-deficiency responsive genes and potential target genes of miRNAs were annotated for gene ontology (GO) terms [78] and categorized into molecular function, cellular component, and biological process categories. GO term enrichment was conducted using single enrichment analysis tool on AgriGo (http://bioinfo.cau.edu.cn/agriGO/) [79]. GO category (http://geneontology.org/) with an adjusted P-value ≤0.05 was regarded as significantly enriched.

### Construction of small RNA libraries and sequencing

Eighteen samples (ZPS, ZMS, ZPR, ZMR, ZRS, and ZRR, each with three biological replicates) were harvested for small RNA library construction and sequencing. Total RNAs were extracted from samples using Trizol reagent (Invitrogen, Carlsbad, CA). Approximately 1.0 μg of total RNA was used to prepare a small RNA library according to the instruction of TruSeq Small RNA Sample Prep Kits (Illumina, San Diego, USA). Single-end sequencing with a length of about 50 bp was performed on an Illumina Hiseq2500 at the LC-BIO (Hangzhou, China). The small RNA sequencing datasets of this study were deposited in NCBI’s Gene Expression Omnibus and are accessible through GEO accession number GSE131003 (https://www.ncbi.nlm.nih.gov/geo/query/acc.cgi?acc=GSE131003).

### Identification of miRNAs and prediction of their target genes

The raw reads were performed on an in-house program ACGT101-miR (LC Sciences, Houston, Texas, USA) to remove adaptor sequences, low-complexity sequences, junk reads, repeat sequences, and the reads that matched the common non-coding RNA families (tRNA, rRNA, snoRNA, and snRNA) deposited in Rfam database (http://www.sanger.ac.uk/software/Rfam). Subsequently, unique sequences with length of 20–24 nucleotide (nt) were mapped to rice miRNA precursors in miRBase 22.0 by BLAST (version 2.6.0) to identify known miRNAs and novel 3p- and 5p- derived miRNAs. Length variation at both 3′ and 5′ ends, and one nt mismatch of the miRNA sequence for the alignment were allowed. The unique sequences mapping to rice mature miRNAs in the precursors were identified as known miRNAs. The unique sequences mapping to the opposite arm of known rice miRNA precursor hairpin were considered to be novel 5p- or 3p- derived miRNA candidates. The remaining sequences were mapped to miRNA precursors of other plant species in miRBase 22.0 (http://www.mirbase.org/) by BLAST (version 2.6.0), and the mapped pre-miRNAs were further aligned with the rice genome to determine their genomic locations. The remaining unmapped sequences were further aligned with the rice genome, and the flanking sequences with a length of 120 nt were collected for secondary structure prediction using RNAfold software (http://rna.tbi.univie.ac.at/cgi-bin/RNAfold.cgi). The criteria for novel miRNA prediction and characterization were based on previous study [80].

The abundance of each miRNA were normalized to the expression of tags per million (TPM) following the formula: normalized expression = actual miRNA count/total count of clean reads*10^6^. To identify Zn deficiency- or Zn resupply- responsive miRNAs, only the miRNAs with a log2 fold change ≥1 or ≤ − 1 and a *p*-value ≤0.05 were considered as significantly differentially expressed miRNAs. To predict potential target genes of Zn-responsive miRNAs, psRNATarget (a plant small RNA target analysis server) (http://plantgrn.noble.org/psRNATarget/) (V2, 2017 release) was employed based on the imperfect complementation between the sequences of miRNAs and their target genes [46].

### Quantitative RT-PCR analysis

Quantitative RT-PCR (qRT-PCR) was performed on a MyiQ Single Color Real-time PCR system (Bio-Rad) as described previously [81]. qRT-PCR analysis of primary miRNA (pri-miRNA) were performed as described previously [59, 82]. The stem-loop sequences were used for primer design, and if no satisfactory primers could be found, stem-loop sequences were elongated by 100 bp of flanking genomic sequences on each side for primer design. Relative expression levels were normalized to that of two internal control genes *OsACTIN1* (*Os03g0718100*) and *OsUBQ2* (*Os02g0161900*) using the Pfaffl method (Ratio = (*E*_target_)^∆CT^_target_^(control-sample)^/(*E*_ref_) ^∆CT^_ref_^(control-sample)^) [83]. The calculated efficiency (E) of all primers was between 1.8 and 2.2. The sequences of primers are listed in Additional file 1: Table S1.

## Supplementary information

**Additional file 1: Table S1.** Primers used for quantitative RT-PCR analysis. **Table S2.** Summary of the reads from RNA sequencing in the 18 libraries. **Table S3.** RNA-seq analysis of rice shoot and root samples under Zn deprivation and Zn resupply. **Table S4.** List of differentially expressed loci in response to 14 d of Zn deprivation in rice shoots. **Table S5.** List of differentially expressed loci in response to 14 d of Zn deprivation in rice roots. **Table S6.** List of differentially expressed loci in response to 3 d of Zn resupply in rice shoots. **Table S7.** List of differentially expressed loci in response to 3 d of Zn resupply in rice roots. **Table S8.** GO representation of the over-represented GO terms in the DEGs regulated by Zn deprivation and/or Zn resupply. **Table S9.** Overview of small RNA sequencing data of the 18 libraries. **Table S10.** Counts and length distribution of total sRNAs and unique sRNAs in this study. **Table S11.** Summary of the detected known and predicted miRNAs in this study. **Table S12.** Differentially expressed miRNAs in response to 14 d of Zn deprivation and/or 3 d of Zn resupply in the roots and shoots. **Table S13.** Potential target genes of the Zn-responsive miRNAs. **Table S14.** Common DEGs identified in this study and earlier studies.

**Additional file 2: Figure S1.** Pearson’s correlation (R-value) of three biological replicates of shoot and root samples for RNA sequencing under control, Zn deficiency, and Zn resupply conditions. Zn plus shoot, ZPS; Zn minus shoot, ZMS; Zn plus root, ZPR; Zn minus root, ZMR; Zn resupply shoot, ZRS; Zn resupply root, ZRR. **Figure S2.** Length distribution of the small RNAs from the small RNA sequencing data in the six samples. Each bar indicates the mean ± SD of three biological replicates. **Figure S3.** Pearson’s correlation (R-value) of three biological replicates of shoot and root samples for small RNA high-throughput sequencing under control, Zn deficiency, and Zn resupply conditions. **Figure S4.** Venn diagram representing the overlap of the Zn-deficiency- and Zn-resupply- responsive miRNAs (a, known and novel miRNAs; b, novel miRNAs) in roots and shoots. **Figure S5.** Predicted secondary structures of 38 Zn-responsive novel miRNAs identified in this study. The red lines indicate the mature miRNA sequences that were found to be differentially expressed under Zn deficiency and/or Zn resupply. The blue lines indicate the miRNA* sequences that were detected by the small RNA sequencing. If no blue line was indicated, it means that no miRNA* sequences were detected by the small RNA sequencing. **Figure S6.** Gene ontology (GO) representation of the overrepresented GO terms of biological processes in the potential target genes of the differentially expressed miRNAs under Zn deficiency and/or Zn resupply. The GO representation was generated using single enrichment analysis (SEA) tool on AgriGO (http://bioinfo.cau.edu.cn/agriGO/) (Fisher’s test, *P* < 0.05, FDR < 0.05). The number in parenthesis represents the FDR value. **Figure S7.** Gene ontology (GO) representation of the overrepresented GO terms of cellular component in the potential target genes of the differentially expressed miRNAs under Zn deficiency and/or Zn resupply. The GO representation was generated using single enrichment analysis (SEA) tool on AgriGO (http://bioinfo.cau.edu.cn/agriGO/) (Fisher’s test, P < 0.05, FDR < 0.05). The number in parenthesis represents the FDR value. **Figure S8.** Gene ontology (GO) representation of the overrepresented GO terms of biological process in the DEGs recovered by Zn resupply in roots and shoots. The GO representation was generated using single enrichment analysis (SEA) tool on AgriGO (http://bioinfo.cau.edu.cn/agriGO/) (Fisher’s test, P < 0.05, FDR < 0.05). The number in parenthesis represents the FDR value.

## Data Availability

The datasets used during the current study are available from the corresponding author on reasonable request.

## Abbreviations

AGO4: Argonaute 4
AO: L-ascorbate oxidase
bZIP: Basic leucine zipper
CCSD: Copper chaperone for superoxide dismutase
CSD: Copper/zinc superoxide dismutase
Cu: Copper
DCL1: Dicer-like 1
DEG: Differentially expressed gene
Fe: Iron
FPKM: Fragments per kilobase of transcript per million mapped reads
GA: Gibberellin
GO: Gene ontology
H3K9: Histone H3 lysine 9
miRNA: microRNA
NAS: Nicotianamine synthase
qRT-PCR: Quantitative RT-PCR
RLM 5′-RACE: RNA ligase-mediated 5′ rapid amplification of cDNA ends
ROS: Reactive oxygen species
SODX: Superoxide dismutaseX
TF: Transcription factor
ZDRE: Zinc deficiency response element
ZIFL: Zinc-induced facilitator
ZIP: ZRT, IRT-related protein
Zn: Zinc
