# Supplementary material for: Integrated analyses of miRNAome and transcriptome reveal zinc deficiency responses in rice seedlings
Source: BMC Plant Biol. 2019 Dec 26;19:585. doi: 10.1186/s12870-019-2203-2 (PMC6933703; doi:10.1186/s12870-019-2203-2)
Supplement: Supplementary file 1 — Additional file 1: Table S1. Primers used for quantitative RT-PCR analysis. Table S2. Summary of the reads from RNA sequencing in the 18 libraries. Table S3. RNA-seq analysis of rice shoot and root samples under Zn deprivation and Zn resupply. Table S4. List of differentially expressed loci in response to 14 d of Zn deprivation in rice shoots. Table S5. List of differentially expressed loci in response to 14 d of Zn deprivation in rice roots. Table S6. List of differentially expressed loci in response to 3 d of Zn resupply in rice shoots. Table S7. List of differentially expressed loci in response to 3 d of Zn resupply in rice roots. Table S8. GO representation of the over-represented GO terms in the DEGs regulated by Zn deprivation and/or Zn resupply. Table S9. Overview of small RNA sequencing data of the 18 libraries. Table S10. Counts and length distribution of total sRNAs and unique sRNAs in this study. Table S11. Summary of the detected known and predicted miRNAs in this study. Table S12. Differentially expressed miRNAs in response to 14 d of Zn deprivation and/or 3 d of Zn resupply in the roots and shoots. Table S13. Potential target genes of the Zn-responsive miRNAs. Table S14. Common DEGs identified in this study and earlier studies. [file 12870_2019_2203_MOESM1_ESM.docx]

**Supplemental Fig. S1** Pearson's correlation (R-value) of three biological replicates of shoot and root samples for RNA sequencing under control, Zn deficiency, and Zn resupply conditions. Zn plus shoot, ZPS; Zn minus shoot, ZMS; Zn plus root, ZPR; Zn minus root, ZMR; Zn resupply shoot, ZRS; Zn resupply root, ZRR.

**Supplemental Fig. S2** Length distribution of the small RNAs from the small RNA sequencing data in the six samples (Zn plus shoot, ZPS; Zn minus shoot, ZMS; Zn plus root, ZPR; Zn minus root, ZMR; Zn resupply shoot, ZRS; Zn resupply root, ZRR). Percentage of each length in the total reads was calculated. Each bar indicates the mean±SD of three biological replicates (n=3).


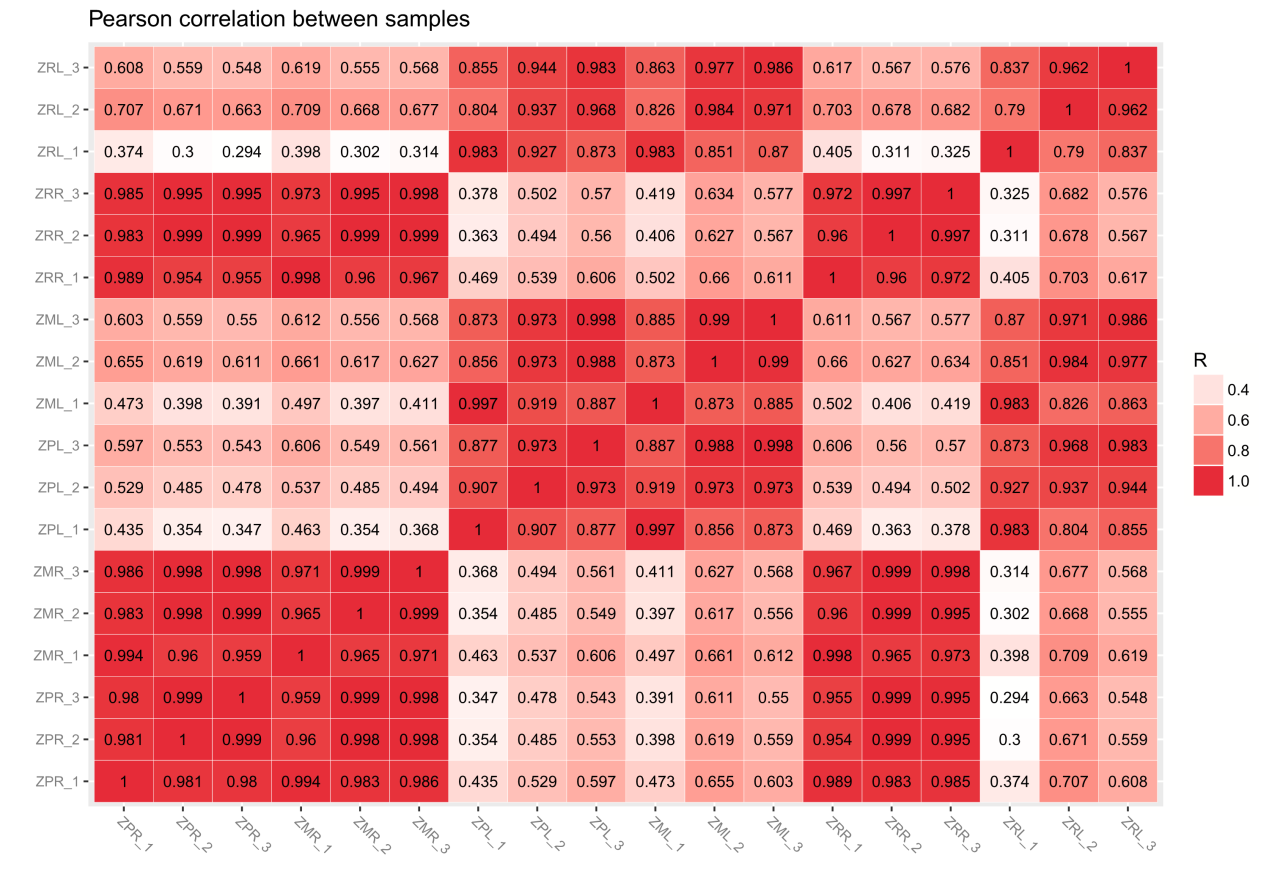


**Supplemental Fig. S3** Pearson's correlation (R-value) of three biological replicates of shoot and root samples for small RNA high-throughput sequencing under control, Zn deficiency, and Zn resupply conditions. Zn plus shoot, ZPS; Zn minus shoot, ZMS; Zn plus root, ZPR; Zn minus root, ZMR; Zn resupply shoot, ZRS; Zn resupply root, ZRR.

**Supplemental Fig. S4** Venn diagram representing the overlap of the Zn-deficiency- and Zn-resupply- responsive miRNAs (a, known and novel miRNAs; b, novel miRNAs) in roots and shoots. Zn plus shoot, ZPS; Zn minus shoot, ZMS; Zn plus root, ZPR; Zn minus root, ZMR; Zn resupply shoot, ZRS; Zn resupply root, ZRR.

continue

continue

continue

**Supplemental Fig. S5** Predicted secondary structures of 38 Zn-responsive novel miRNAs identified in this study. The red lines indicate the mature miRNA sequences that were found to be differentially expressed under Zn deficiency and/or Zn resupply. The blue lines indicate the miRNA* sequences that were detected by the small RNA sequencing. If no blue line was indicated, it means that no miRNA* sequences were detected by the small RNA sequencing.

**Supplemental Fig. S6** Gene ontology (GO) representation of the overrepresented GO terms of biological processes in the potential target genes of the differentially expressed miRNAs under Zn deficiency and/or Zn resupply. The GO representation was generated using single enrichment analysis (SEA) tool on AgriGO (http://bioinfo.cau.edu.cn/agriGO/) (Fisher’s test, P < 0.05, FDR < 0.05). The number in parenthesis represents the FDR value.

**Supplemental Fig. S7** Gene ontology (GO) representation of the overrepresented GO terms of cellular component in the potential target genes of the differentially expressed miRNAs under Zn deficiency and/or Zn resupply. The GO representation was generated using single enrichment analysis (SEA) tool on AgriGO (http://bioinfo.cau.edu.cn/agriGO/) (Fisher’s test, P < 0.05, FDR < 0.05). The number in parenthesis represents the FDR value.

**Supplemental Fig. S8** Gene ontology (GO) representation of the overrepresented GO terms of biological process in the DEGs recovered by Zn resupply in roots and shoots. The GO representation was generated using single enrichment analysis (SEA) tool on AgriGO (http://bioinfo.cau.edu.cn/agriGO/) (Fisher’s test, P < 0.05, FDR < 0.05). The number in parenthesis represents the FDR value.
